# Evaluation of working conditions at a central sterile services department in northern Brazil

**DOI:** 10.47626/1679-4435-2021-623

**Published:** 2021-12-30

**Authors:** Valéria Moreira da-Silva, Daniela Oliveira Pontes, Priscilla Perez da Silva Pereira, Janne Cavalcante Monteiro, Mônica Nascimento Cruz

**Affiliations:** 1 Departamento de Enfermagem, Fundação Universidade Federal de Rondônia, Porto Velho, RO, Brazil.; 2 Departamento de Medicina, Fundação Universidade Federal de Rondônia, Porto Velho, RO, Brazil.

**Keywords:** working conditions, sterilization, occupational health, ergonomics, condições de trabalho, esterilização, saúde do trabalhador, ergonomia

## Abstract

**Introduction:**

Environmental conditions and the work process in the Central Sterile Services Department expose workers to many hazards.

**Objectives:**

To analyze the working conditions that have an influence on the overall health of workers at a Central Sterile Services Department of a hospital in northern Brazil.

**Methods:**

This study used employed the ergonomic analysis of work. Three data collection tools were applied to characterize sociodemographic and occupational profiles. Furthermore, physical environmental hazards were analyzed using specific equipment. Descriptive and bivariate analyses were performed in Stata^®^13 software.

**Results:**

Thirty-five workers participated in the study, most of whom were women, aged above 40 years, and worked as nursing technicians. It became evident that workers were exposed to inappropriate lighting and to noises coming from autoclaves, from the central air conditioning, and from the use of medicinal gas. The risks for musculoskeletal injuries on spine, shoulders, and lower limbos were specifically related to overload resulting from the posture maintained for long periods. Female sex was associated with severe or excruciating pain” (p = 0.04).

**Conclusions:**

Sound, mechanical, and visual hazards were found. Most participants complained of musculoskeletal pain, and the cervical spine was the site with the most frequent reports of excruciating pain. These results point out to the need of a policy to improve the quality of work in the health care unit studied; however, these findings may also bring light to the problem in other institutions that have a physical structure similar to that found in this research.

## Resumo

**Introdução:**

As condições ambientais e o processo de trabalho no Centro de Material e Esterilização expõem os trabalhadores a diversos riscos.

**Objetivos:**

Analisar as condições de trabalho que influenciam a saúde global dos trabalhadores do Centro de Material e Esterilização de um hospital do norte do Brasil.

**Métodos:**

Utilizou-se a metodologia de análise ergonômica do trabalho. Foram aplicados três instrumentos de coleta de dados para caracterizar os perfis sociodemográfico e ocupacional. Além disso, foram analisados os riscos físico-ambientais utilizando equipamentos específicos. As análises descritiva e bivariada foram realizadas no *software* Stata^®^13.

**Resultados:**

Participaram do estudo 35 trabalhadores, sendo a maioria mulheres, com idade acima de 40 anos e técnicos de enfermagem. Evidenciou-se que os trabalhadores eram expostos a iluminância inadequada e ruídos originados das autoclaves, da central do ar-condicionado e do uso do gás medicinal. Os riscos para lesões musculoesqueléticas na coluna, no ombro e nos membros inferiores especificamente se relacionam à sobrecarga exigida pelas posturas assumidas por longos períodos. O sexo feminino foi associado com dor do tipo “bastante ou extrema” (p = 0,04).

**Conclusões:**

Foram encontrados riscos sonoros, mecânicos e visuais. A maioria dos participantes apresentou queixas de dores musculoesqueléticas, sendo a coluna cervical o local de maior frequência de dor do tipo extrema. Esses resultados apontam para a necessidade de uma política de melhorias da qualidade do trabalho na unidade estudada; porém, esses achados também podem trazer luz à problemática em outras instituições que possuam estrutura física semelhante à encontrada nesta pesquisa.

## INTRODUCTION

For the developed work to reach its goals, workers need to have appropriate conditions to perform their activities. Therefore, in the relationship between people and work, it is important to know the conception of tasks and inherent conditioning factors that may impair worker’s health.

When describing the activities developed in a Central Sterile Services Department (CSSD), one should consider the specific peculiarities of this department. The CSSD is a unit whose main objective is to dispense health products (HPs) that are safe for use in health care units and that do not cause damage to users.^1^ Individuals working at the CSSD are frequently exposed to physical, biological, chemical, and ergonomic risks and to external conditions, such as repetitive physical exertion, exhausting working hours, and accelerated work pace. It bears emphasizing that these conditions may interfere with the quality of the performed activities and with workers’ health.^2,3^

Previous studies indicate that some CSSDs may present inadequate physical infrastructure, small isolated spaces, precarious ventilation systems, and other conditioning factors that may worsen workers’ health, cause illness, and increase the risk of accidents in the workplace.^4,5^ Ergonomics studies are important to understand environmental risks to which workers are exposed and subsidize improvements in this environment, leading to lower rates of absenteeism and improvements in the work process and, more importantly, contributing for a better health status among workers. Investigations on CSSDs are still scarce in the literature; therefore, the aim of this study is to analyze the working conditions of health care professionals who work at the CSSD of a public general hospital in the state of Rondônia, Brazil, and to assess the extent to which these conditions interfere with their health.

## METHODS

This is a cross-sectional, descriptive, quantitative study that employed the ergonomic analysis of work. This method uses several techniques, such as systematic and non-systematic observation, behavioral recording, questionnaire-based investigation, and assessment of physical environmental hazards.^6^

The study was conducted in the municipality of Porto Velho, capital city of the state of Rondônia, Brazil. In 2017, this municipality had 10 CSSDs, four of which were located in public hospitals. The CSSD selected for this study operates in a large institution that is a referral center for tertiary care in the state, representing the largest public hospital in the state. The institution has 610 beds and 2,186 employees; furthermore, it serves users of the Brazilian Unified Health System (Sistema Único de Saúde, SUS) from 52 municipalities in the state of Rondônia, as well as from the neighboring states of Amazonas and Acre and from the neighboring country of Bolivia.^7^

The surgical procedures performed in the study hospital are of medium and high complexity, with approximately 1,000 operations per month, in addition to diagnostic procedures and health care in clinics and adult and neonatal intensive care units. The CSSD of this hospital is classified as class II according to Resolution no.15 of the Brazilian National Health Surveillance Agency (Agência Nacional de Vigilância Sanitária, ANVISA), which means that it is allowed to process critical, semi-critical, and non-critical materials of complex and non-complex conformation.^8,9^

The study population consisted of nurses and nursing assistants/technicians, totaling 41 workers. All professionals who were present in the unit during the data collection period were included. Exclusion criteria were being on sick leave, vacation, or bonus leave.

Data collection was conducted from March 2016 to April 2017. The research included assessment of physical environmental hazards, non-participant systematic observation of an actual work situation, and a two-part questionnaire, which was administered by the participants themselves. The first part covered participants’ sociodemographic, occupational, and lifestyle aspects and contained 19 open and close questions based on the instrument proposed by Santos;^10^ whereas the second part approached pain complaints after the work shift on a body segment map according to the Corlett and Manenica Diagram.^11^ Pain was assessed using numbers from 1 to 5 points, ranging from: 1) no pain; 2) mild pain; 3) moderate pain; 4) severe pain; 5) excruciating pain.

The assessment of physical environmental hazards (noise, lighting, and temperature) was performed by an occupational engineer. Measurements were taken in the morning, based on the period of greater activity in the department (from 9 a.m. to noon). Measurements were obtained for 5 minutes during the performance of activities and did not interfere with these activities. Assessment of noise levels was conducted using the measuring equipment Termo-Higro-Decibel-Luxim, Instrutherm brand, model THDL-400 with calibration certificate no. 6314/15 and complied with the recommendations of Regulatory Standard (Norma Regulamentadora, NR) 17 and of ABNT NBR-10152 (NB-95), which provide that measures should be obtained close to worker’s ear during the performance of activities of preparation and packing on the counters and handling the trolley that carries surgical boxes and packages from the sterilization area (autoclaves room). Lightening and the temperature were also measured by the mentioned equipment in compliance with the following regulatory standards, respectively: NBR/CIE 8995, NBR 5413/1992 and NR 17.^12-16^

Initially, descriptive analysis was conducted using absolute and relative frequencies to characterize workers’ variables and pain distribution into body segments. Furthermore, a bivariate analysis was performed between the dependent variable pain (no pain, mild pain, or moderate pain vs. severe pain or excruciating pain) and sociodemographic, occupational, and lifestyle characteristics using the Pearson chi-square or the Fisher exact test, considering a level of statistical significance of p < 0.05. All analyses were conducted in the Stata 13 software.

This study was approved by the Research Ethics Committee of Fundação Universidade Federal de Rondônia (UNIR) under opinion 1.849.750. The ethical principles of Resolution no. 466, of December 12, 2012, of the National Health Council were followed.^17^

## RESULTS

All employees at the CSSD were invited to participate in this study. Thirty-nine nursing assistants and/or technicians and six nurses were interviewed. Six workers were excluded because they were on sick leave or vacation during the data collection period. Workers’ mean age was 48 years (standard deviation 9.43). Most workers were female, worked as nursing assistant and/or technician, had a family income of up to four minimum wages, had graduated more than 10 years ago, and had been working at the department for more than 5 years. Almost half of the professionals worked in the area of preparation and sterilization, and most did not perform roles in the CSSD other than the assigned one, with an on-duty work schedule of up to 40 hours per week, with no other employment relationship (Table 1).

**Table 1:** Relationship between workers’ characteristics and occurrence of pain in the Central Sterile Services Department (CSSD), Porto Velho, state of Rondônia, Brazil, 2017 (n = 35)

Variable	Frequency		No pain, mild pain, or moderate pain		Severe or excruciating pain		p-value
	n	%	n	%	n	%	
Gender							0.04
Female	27	77.1	6	22.2	21	77.7	
Male	8	22.9	5	62.5	3	37.5	
Professional category							0.64
Assistant technician*	29	82.9	10	34.5	19	65.5	
Nurse	6	17.1	1	16.7	5	83.3	
Age (years)							0.27
26 to 30	2	5.7	1	50.0	1	50.0	
31 to 35	2	5.7	0	0.0	2	100.0	
36 to 40	5	14.3	1	20.0	4	80.0	
41 to 45	6	17.1	4	66.7	2	33.3	
46 to 50	2	5.7	1	50.0	1	50.0	
Above 50	18	51.4	4	22.2	14	77.8	
Family income in minimum wages^†^							0.06
0-4	21	60.0	10	47.6	11	52.4	
5-10	9	25.7	1	11.1	8	88.9	
> 10	5	14.3	0	0.0	5	100.0	
Time in profession (years)							0.49
0-4	3	8.6	0	0.0	3	100.0	
5-9	6	17.1	1	16.7	5	83.3	
≥ 10	26	74.3	10	38.6	16	61.4	
Time working at the CSSD (years)							0.30
0-4	10	28.6	5	50.0	5	50.0	
5-9	14	40.0	4	28.6	10	71.4	
≥ 10	11	31.4	2	18.2	9	81.8	
Processing stage in which the worker is involved							0.57
1	10	28.6	2	20.0	8	80.0	
2	17	48.6	7	41.2	10	58.8	
3	8	22.8	2	25.0	6	75.0	
Other role in the CSSD							0.49
No	30	85.7	10	33.3	20	66.7	
Yes	5	14.3	1	20.0	4	80.0	
Work schedule							0.91
On-duty	29	82.9	9	31.0	20	69.0	
Day laborer	6	17.1	2	33.3	4	66.7	
Working hours at the department (hours)							0.4
≤ 40	33	94.9	10	30.3	23	69.7	
> 40	2	5.1	1	50.0	1	50.0	
More than one employment relationship							0.65
No	21	60.0	6	28.6	15	71.4	
Yes	14	40.0	5	35.7	9	64.3	
Period of rest break							0.34
Morning	12	34.3	5	41.7	7	58.3	
Afternoon	9	25.7	1	11.1	8	88.8	
Evening	14	40.0	5	35.7	9	64.3	
Duration of rest break (minutes)							0.95
≤ 30	22	62.9	7	31.8	15	68.2	
> 30	13	37.1	4	30.8	9	69.2	
Regular practice of physical exercise^‡^							0.27
No	19	54.3	4	21.1	15	78.9	
Yes	16	45.7	7	43.7	9	56.3	
Smoking status							0.36
Never smoked	25	71.4	9	36.0	16	64.0	
Former smoker	8	22.9	1	12.0	7	88.0	
Smoker	2	5.7	1	50.	1	50.	
Body mass index^§^							0.82
Underweight	-	-	-	-	-	-	
Normal weight	10	28.6	3	30.0	7	70.0	
Overweight	15	42.9	6	40.0	9	60.00	
Obesity grade I	8	22.9	2	25.0	6	75.00	
Obesity grade II	-	-	-	-	-	-	
Obesity grade III	2	5.6	0	0.0	2	100.00	

* Nursing assistant or technician.

† Minimum wage in the year 2017 (BRL 937.00).

‡ Three times a week, duration from 30 to 40 minutes.

§ According to World Health Organization (2002).^19^

The time of resting break was shorter than 30 minutes, and distribution of the period for this break varied according to the shift period. A little more than a half of participants did not perform physical exercise, 22.9% were former smokers, and 5.7% still smoked. Most respondents were classified into overweight or obesity.

When assessing the relationship between workers characteristics and occurrence of pain (Table 1), we found a statistically significant difference only for gender, with the female gender being associated with “severe pain” or “excruciating pain” (p = 0.04; Table 1). No differences were observed for the remaining sociodemographic, occupational, and lifestyle characteristics with regard to the manifestation of pain classified as “severe” or “excruciating”.

Table 2 presents the structural characteristics of the CSSD and its physical environmental hazards. Temperature and lighting varied among workplaces. There were benches and desks with non-standardized heights and that did not suit the needs of workers, whose mean height was 1.60 m (minimum 1.48 m and maximum 1.80 m). In addition to these items, there was also lack of chairs with adjustable height, backrest, and footrest (Table 2).

**Table 2:** Results for physical environmental hazards at work areas in the Central Sterile Services Department of a public general hospital, Porto Velho, state of Rondônia, Brazil, 2017

Processing stage	Work area/number of professionals per shift	Size	Tempe­rature (ºC)	Lighting (lux)	Noise dB (A)	Furniture	Other hazards observed
Reception and cleaning	Area of reception of HPs coming from the SC/two workers	Area of 5.01 m^2^, perimeter of 9.34 m, CH: 2.96 m	27.90	49.00	58.00	Bench (94 cm in height and 147 cm in width), a sink with a bench, soap dispenser, and paper towel, one aluminum trolley with a door measuring 81 cm in height and 90 cm in width, no chairs or seats.	No appropriate adherence to the use of PPE by workers.
	Area of reception from clinics and of cleaning of HPs/ same workers of reception from the SC	Area of 24.30 m^2^, perimeter of 19.73 m, CH: 2.96 m.	18.00	180.00-275.00	87.00*	Sinks with 40 cm in depth, stainless steel bench, and faucets. Benches with 92 cm of height, a cabinet with doors measuring 1.86 cm in height and 100 cm in width. One plastic chair with no adjustable height or backrest. No foot or leg rest. Magnifying glasses as a device to aid manual inspection. Ultrasound cleaners. Compressed air cylinder.	No appropriate adherence, by workers, to the use of PPE recommended in this area (nitrile gloves, impermeable boots, long-sleeved apron). Ergonomic hazards - position in the activity and excess weight. Biological, mechanical, chemical hazards.
Preparation and sterilization	Area of preparation of HPs/two workers	Area of 57.37 m^2^, perimeter of 34.83 m, CH: 2.96 m	17.00	77.50-113.40	78.00-79.20	Three desks - two with 98 cm in height and 100 cm in width by 320 cm in length and one with 98 cm in height and 100 cm in width by 400 cm in length with wired baskets. Thermosealing machines. Three chairs with no adjustable backrest, defects in height adjustment and no foot and leg rest. Compressed air pistols.	No use of lumbar corset to handle packages. Ergonomic risks - position in the activity and excess weight.
	Area of sterilization of HPs/one worker	Area of 31.31 m^2^, perimeter of 22.58 m, CH: 2.96 m	27.00	21.00	67.00	Autoclaves, transportation trolleys did not have handles to push them, and their wheels did not roll properly. Non-ergonomic chair, with no adjustable height or backrest, and no footrest.	Workers with sterilized and contaminated materials pass by each other in the aisle. Lack of appropriate PPE to handle contaminated material by all workers who pass by.
Storage and distribution	Area of storage and distribution of HPs/one worker	Area of 6.40 m^2^, perimeter of 10.39 m, CH: 2.96 m	26.50	26.10 (without turning on artificial light); 280 (with artificial light turned on)	62.00	Two staircases with two steps; one table with 70 cm in height and 67 cm in width; non-ergonomic chair, no adjustable backrest and height, no footrest. Two shelves with 180 cm in height and 50 cm in width, a cabinet with 186 cm in height and 100 cm in width, a shelf with 197 cm in height and 93 cm in width, two cabinets with 195 cm in height and 93 cm in width. Shelves having 20 cm of distance to the floor and 45 cm to the ceiling, but the distance from the walls does not comply with the recommended 5-cm distance. One plastic chair with no height adjustment or backrest. No foot or leg rest.	No use of lumber corset to handle packages.

* Illuminance in this area should be 300lux as recommended by NBR/CIE 8995.

CH = ceiling height; HP = health product; PPE = personal protection equipment; SC = surgical center.

Routine activities performed in the CSSD, in addition to ergonomic inadequacies, may contribute to the occurrence of physical discomforts. Table 3 presents the frequency of professionals who reported some degree of pain after the work shift. Of the 26 body segments considered, the right shoulder was the site with the greatest frequency of pain complaints (74.29%). A total of 14 segments presented with reports of unbearable excruciating pain, of which the most frequent was cervical spine (n = 3; 8.57%). Hip (60%), left hand (65.71%), right thigh (68.57%), and left thigh (77.14%) were the sites where most respondents reported not having any pain (Table 3).

**Table 3:** Distribution of frequency of pain on workers’ body segments with regard to the stages of Processing of Health Products in the Central Sterile Services Department, Porto Velho, state of Rondônia, Brazil, 2017

Body segments	No pain		Mild pain		Moderate pain		Severe pain		Excruciating pain	
	n	%	n	%	n	%	n	%	n	%
Neck	15	42.86	5	14.29	6	17.14	7	20.00	2	5.71
Cervical spine	11	31.43	6	17.14	7	20.00	8	22.86	3	8.57
Right shoulder	9	25.71	5	14.29	12	34.29	8	22.86	1	2.86
Left shoulder	15	42.86	3	8.57	9	25.71	6	17.14	2	5.71
Thoracic spine	15	42.86	9	25.71	4	11.43	7	20.00	0	0.00
Right arm	14	40.00	3	8.57	11	31.43	7	20.00	0	0.00
Left arm	17	48.57	2	5.71	9	25.71	7	20.00	0	0.00
Lumbar spine	14	40.00	5	14.29	8	22.86	7	20.00	1	2.86
Right forearm	16	45.71	10	28.57	7	20.00	2	5.71	0	0.00
Left forearm	19	54.29	8	22.86	6	17.14	2	5.71	0	0.00
Low back	16	45.71	4	11.43	7	20.00	8	22.86	0	0.00
Right wrist	16	45.71	9	25.71	6	17.14	4	11.43	0	0.00
Left wrist	19	54.29	7	20.00	5	14.29	3	8.57	1	2.86
Hip	21	60.00	5	14.29	6	17.14	3	8.57	0	0.00
Right hand	20	57.14	7	20.00	5	14.29	3	8.57	0	0.00
Left hand	23	65.71	5	14.29	4	11.43	3	8.57	0	0.00
Right thigh	24	68.57	6	17.14	2	5.71	2	5.71	1	2.86
Left thigh	27	77.14	4	11.43	1	2.86	2	5.71	1	2.86
Right knee	18	51.43	6	17.14	4	11.43	6	17.14	1	2.86
Left knee	16	45.71	6	17.14	7	20.00	4	11.43	2	5.71
Right leg	14	40.00	5	14.29	11	31.43	4	11.43	1	2.86
Left leg	15	42.86	5	14.29	10	28.57	4	11.43	1	2.86
Right ankle	16	45.71	6	17.14	4	11.43	8	22.86	1	2.86
Left ankle	17	48.57	5	14.29	6	17.14	6	17.14	1	2.86
Right foot	12	34.29	5	14.29	9	25.71	8	22.86	1	2.86
Left foot	13	37.14	5	14.29	9	25.71	7	20.00	1	2.86

## DISCUSSION

In this research, most participants were women, and the variable gender was the only one that had a statistical significance when assessing its association with severe or excruciating pain. This finding corroborates that of a study conducted in 2017 at a CSSD of a university hospital in the state of Maranhão, Brazil, with 82 professionals. Of these, 72% were female and, in the assessment of quality of life, the most affected domain was pain.^18^

The predominant age group in the present research was that above 50 years, and most participants had been working at the CSSD for more than 5 years. Since it was a department where the activities developed require great physical exertion, changes in the musculoskeletal system resulting from the aging process, such as bone density loss, loss of muscle strength and size, and cartilage degeneration, predispose professionals to inflammatory processes, possible musculoskeletal injuries, and pain.^19^

With regard to family income, most professionals in the study had a family income of up to four minimum wages and on-duty work schedule. A total of 40% of participants presented more than one employment relationship. A study conducted in 2013 in a university hospital in city of Rio de Janeiro, Brazil, with a sample of 34 professionals working at a CSSD, found that 70.59% of respondents had another job because of the low wages that predominate in health services, especially in the nursing profession.^3^

In the present study, most workers were overweight or obese, and less than a half performed physical exercise. This finding may interfere with the onset of musculoskeletal pain, considering the pressure on bones and joints caused by excessive weight, associated with postures maintained for long periods when working at the CSSD.^20^

With regard to the structure of the CSSD, we found areas with temperatures of up to 28.8 ºC, whereas the optimal temperature ranges from 18 to 25ºC in order not to compromise sterilization of HPs stored in shelves and cabinets. Moreover, thermal discomfort has a direct influence on productivity and increases the occurrence of accidents in the workplace. Heath is considered the most reported physical hazard and the leading cause of discomfort in the workplace.^5,9,21-23^

Lighting in health care environments is essential for the performance of a high-quality work, with less physical exertion. Insufficient lighting is a risk factor for diseases and accidents in the workplace. Illuminance inside the CSSD should be of 300 lux, as recommended by NBR/CIE 8995 and by NBR 5413/1992, and may reach up to 750 lux, since it is a work space that requires visual accuracy.^15,16^

Some areas of the CSSD had noise levels above the recommended, which is 65 dB (A) as a maximum. This finding was also observed in a study conducted in a hospital of the state of São Paulo, Brazil, which showed that the main source of noise was using compressed air to dry products; however, our study had noise levels higher than those found in the aforementioned study.^24,25^

With regard to furniture, the height of the bench in the area of reception and cleaning of HPs was 92 and 94 cm, whereas the recommended height for tasks that require more concentration is from 100 to 110 cm for men and from 95 to 105 cm for women. Sink depth was within the recommended range by RDC/ANVISA no. 15, but they may lead workers to lean the torso forward, causing spine tension and contraction of muscles and ligaments of the chest. There were chairs available for use during work and for rest, but they did not comply with the parameters of NR 17-Ergonomics, which recommends chairs with height adjustable backrest, in the anteroposterior direction and with forma slightly adjusted to the body to protect the low back region.^9,13,26,27^

The work at the CSSD is performed by means of repetitive activities executed for long periods. In our study, of the 26 body segments studied, 17 were reported as painful sites for at least 50% of workers. The following regions stood out as those with the greater pain intensity: neck, cervical spine, right and left shoulders, left wrist, and left knee. These results were similar to those found in a study conducted in the state of Paraná, Brazil, with 30 professionals, in which 54% of nurses presented with low back pain in the last 12 months; of these, 10% were of severe intensity.^28^

Remaining in the standing position without moving for long periods and leaning the torso forward to prepare packages and surgical boxes or clean the material may lead to changes in the axis of the spine and in intradiscal pressure, causing discomfort and pain on neck and on shoulders. Pressing a compressed-air pistol against the instruments to dry them may cause stress on the joints of shoulders, arms, writs, and hands. Adopting a forced posture to catch a package on the shelf, which may lead to sprain of ligaments and joints of arm and shoulders and to tension on the cervical spine, explains the complaints of pain and discomfort on neck, cervical spine, and shoulders. Moreover, the activity of pushing the rack from within the autoclave with the sterilized load requires muscle strength and generates mechanical tension on muscles and joints, causing muscle fatigue and repetition strain injuries.^6,27,29,30^

The limitation of this study was the fact that it analyzed only one CSSD. However, since this service is provided in most hospitals in Brazil, we understand that our findings may contribute for the reflection on working conditions and on the occupational hazards to which professionals are exposed in this health care unit.

## CONCLUSIONS

In the CSSD assessed, professionals were exposed to sound, mechanical, and visual hazards. Most participants complained of musculoskeletal pain, and the cervical spine was the site with the most frequent reports of excruciating pain. The occurrence of more excruciating pain was associated with the female sex, the predominant gender in the department. These results point out to the need of a policy to improve the quality of work in the health care unit studied, moreover, they may also bring light to the problem in other institutions with a physical structure similar to that found in the present research. Assessing occupational hazards in CSSD in the most different realities contribute to the discussion on workers’ health, but there is still a gap to be investigated about the impact of environmental inadequacies and worker’s complaint of pain on the quality of HP processing.
